# Challenges in Dengue Vaccines Development: Pre-existing Infections and Cross-Reactivity

**DOI:** 10.3389/fimmu.2020.01055

**Published:** 2020-06-16

**Authors:** Abdullah M. Izmirly, Sana O. Alturki, Sawsan O. Alturki, Jennifer Connors, Elias K. Haddad

**Affiliations:** ^1^Division of Infectious Diseases and HIV Medicine, Department of Medicine, Drexel University College of Medicine, Philadelphia, PA, United States; ^2^Department of Microbiology and Immunology, Drexel University College of Medicine, Philadelphia, PA, United States; ^3^Department of Medical Technology, Faculty of Applied Medical Sciences, King Abdulaziz University, Jeddah, Saudi Arabia

**Keywords:** dengue (DENV), adenosine deaminase (ADA), antibody dependent enhancement (ADE), challenges of vaccine development, T follicular helper cells (Tfh), cross-reactivity

## Abstract

Dengue is one of the most frequently transmitted mosquito-borne diseases in the world, which creates a significant public health concern globally, especially in tropical and subtropical countries. It is estimated that more than 390 million people are infected with dengue virus each year and around 96 million develop clinical pathologies. Dengue infections are not only a health problem but also a substantial economic burden. To date, there are no effective antiviral therapies and there is only one licensed dengue vaccine that only demonstrated protection in the seropositive (Immune), naturally infected with dengue, but not dengue seronegative (Naïve) vaccines. In this review, we address several immune components and their interplay with the dengue virus. Additionally, we summarize the literature pertaining to current dengue vaccine development and advances. Moreover, we review some of the factors affecting vaccine responses, such as the pre-vaccination environment, and provide an overview of the significant challenges that face the development of an efficient/protective dengue vaccine including the presence of multiple serotypes, antibody-dependent enhancement (ADE), as well as cross-reactivity with other flaviviruses. Finally, we discuss targeting T follicular helper cells (Tfh), a significant cell population that is essential for the production of high-affinity antibodies, which might be one of the elements needed to be specifically targeted to enhance vaccine precision to dengue regardless of dengue serostatus.

## Epidemiology of Dengue Infection

Dengue is a global health threat in tropical and subtropical countries with a vast number of dengue infections that has been estimated to be more than 390 million cases annually. Among them, ~96 million people develop clinical pathologies (1). In 2019, there were many cases of dengue infection reported worldwide, of which more than 3 million cases were confirmed by the Pan American Health Organization (PAHO). The majority of the cases were reported in Brazil with an estimated 1.5 millions in 2019 (PAHO). This accounted for more than 10-fold increase compared with the year before. In addition to South American countries, dengue infection occurs in multiple countries in Asia and Southeast Asia, including Bangladesh, Malaysia, the Maldives, and the Philippines, where dramatic increases in dengue infection cases are on the rise. Countries in the Indian Ocean, Australia, and the Pacific have reported many dengue cases as well. A complete list of countries with dengue infection is found in Table 1. Dengue infections are not only a health problem but also a huge economic burden that has been estimated with a total annual burden of ~$5.71 billion dollars in 2016 (2). This economic burden has risen dramatically from the estimate of 2013, which was $1.51 billion dollars and is likely to continue to rise yearly (2). Thus, there is an urgent need to develop a dengue vaccine and this exemplified by the international collaborative efforts from many world health organizations and federal institutions.

**Table 1:** List of total dengue cases by year and country.

**Country**	**Cases 2019**	**Cases 2020**	
**Indian Ocean**			
Mayotte	9	904	French Regional Health Agency (ARS)
Reunion	3,048	353	French Regional Health Agency (ARS)
**Pacific Islands Countries and Australia**			
Australia	1,038	54	Department of Health, Australia
French Polynesia	3,496	168	Center for Occupational Health and Public Safety, French Polynesia
New Caledonia	319	11	Network of sentinel physicians, New Caledonia
**Asia**			
Cambodia	56,000	330	Ministry of Health, Cambodia
China	20,000	268	National Health Commission, China
Viet Nam	370,702	NA	General Department of Preventative Medicine, Ministry of Health, Viet Nam
Lao PDR	2,300	285	National Centre for Laboratory and Epidemiology, Ministry of Health, Lao PDR
Malaysia	100,803	18,473	Department of Health, Malaysia
The Philippines	27,245	15,817	
Singapore	2,506	1,729	Communicable Diseases Division, Ministry of Health, Singapore
Thailand	136,000^**^	2,097	Bureau of Epidemiology, Department of Disease Control, Ministry of Public Health
Indonesia	110,000^***^	NA	WHO
Sri Lanka	99,120	14,730	Epidemiology Unit of the Ministry of Health, Sri
**Americas and the Caribbean**			
North America	1,158	35	Pan American Health Organization (PAHO)
Central America Ithsmus and Mexico	672,168	30,460	
Andean Subregion	185,320	56,077	
Southern Cone (incl Brazil)	2,241,974	273,565	
Latin Caribbean	23,472	1,267	
Non-Latin Caribbean	16,557	3,401	
Europe	NA	NA	European Centre for Disease Prevention and Control

^*^No official updates for Bangladesh, the Maldives, and India.

** *No official update since November 2019*.

*** *No official update since October 2019*.

*All numbers reported for 2020 are as of February 5, 2020*.

## Clinical Manifestations of Dengue Infection

**Figure 1:**
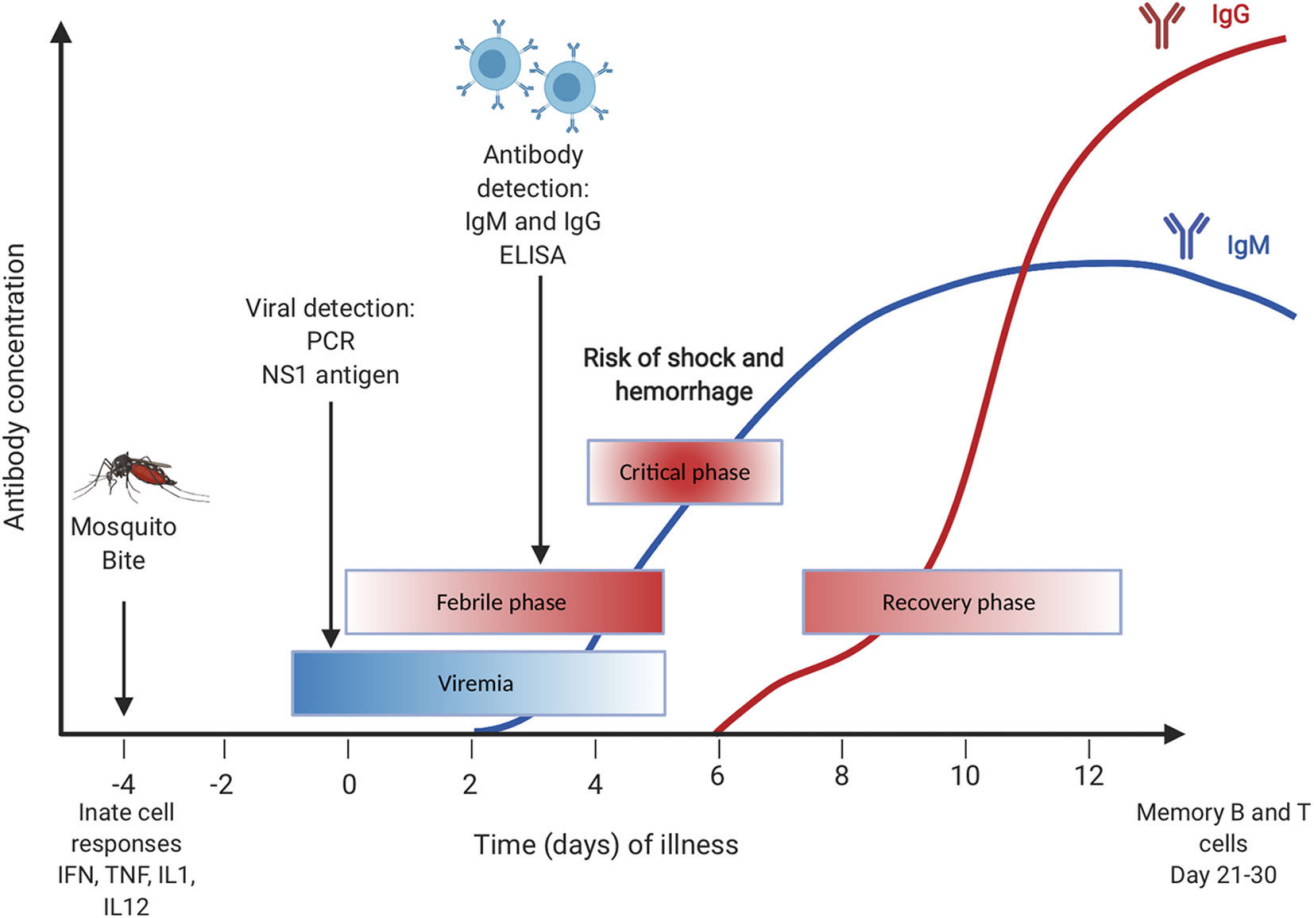
Primary dengue infection with timing of diagnostic testing.

There are three phases of dengue infection: the febrile phase, the critical phase, and the recovery phase. The febrile phase is the initial phase and usually begins with a high fever that appears with several flu-like symptoms, including vomiting, headaches, myalgia, and joint pain. This phase lasts for about a week and most people recover without further complications (3). The critical phase, or the life-threatening phase, is where most of the severe symptoms, such as internal bleeding and plasma leakage, occur. During the recovery phase, the vascular permeability returns to normal, and symptoms start to subside rapidly (3) (Figure 1). In general, the most severe form of the disease is developing dengue hemorrhagic fever (DHF) and dengue shock syndrome (DSS) both of which are associated with antibody-dependent enhancement (ADE) (4, 5). ADE occurs when an antibody that is generated previously to one of the four serotypes binds but does not neutralize another dengue serotype. This binding then facilitates the entry of viruses via FC receptors which results in increased viremia (5).

## Virology of Dengue Virus

Dengue virus (DENV) is an arthropod-borne pathogen that infects humans by a bite of an infected mosquito (6). Two types of mosquitos can be infected by the dengue virus: *Aedes aegypti* or *Aedes albopictus* (1). Dendritic cells (DCs) and macrophages are primarily targeted by the virus in the first days of dengue virus infections (7). There is no human-to-human transmission, and the virus is only transmitted through mosquitos when taking blood from a viremic individual. Viremia and systemic infection can be accomplished due to lymphotropic characteristics of the virus in which the DENV infects skin-draining lymph nodes (dLNs) and the cells that traffic into them, such as DCs and monocytes (8).

DENV is an enveloped virus that consists of a positive-stranded RNA that belongs to the Flavivirus genus of the Flaviviridae family. When the virus is matured, it encompasses three structural proteins namely the nucleocapsid (C), envelope (E), and membrane (M) and seven non-structural proteins (NS1, NS2A, NS2B, NS3, NS4A, NS4B, and NS5) (9). These play significant roles in virus genome replication, immune system evasion and modulation, virion assembly, and viral genome synthesis (10). DENV has four antigenically distinct serotypes (DENV1-4) that share up to 65% of their viral genome (11, 12). The differences in the serotypes create a great challenge for dengue vaccine development. The dengue virus enters the host cell through various internalization pathways as clathrin-dependent receptor endocytosis when bound to a cognate receptor. During natural infection, DENV primarily infects cells bearing C-type lectin receptors on mononuclear phagocyte lineage cells like monocytes, DCs, and macrophages. Such C-type lectin receptors are, for example, DC-specific intracellular adhesion molecule 3 (ICAM-3) and grabbing non-integrin (DC-SIGN, CD209) (6). In secondary infections, DENV depends on the pre-existing antibodies to be taken up by target Fcγ receptor-bearing cells to enter the host cells. Upon entering the cell by endocytosis, DENV can escape the endosome, due to a pH-dependent conformational change, and release its genome to the cytoplasm (13). Following translation of the structural and non-structural proteins, the capsid and the genome associate together to form a nucleocapsid in the cytoplasm. Nucleocapsids are directed by an unknown mechanism to the ER and bud into the lumen of the ER to acquire the bilipid membrane coated with prM/M proteins and E proteins (6, 13). This will form a spike-like shaped immature virus which will then be directed to the Golgi apparatus for additional structural changes in prM. The slightly acidic pH of the trans-Golgi network (TGN) and the presence of the host cell endoprotease furin enable the cleavage of prM to generate a smooth marble-like shaped mature virion-associated M and a soluble peptide (14).

**Figure 2:**
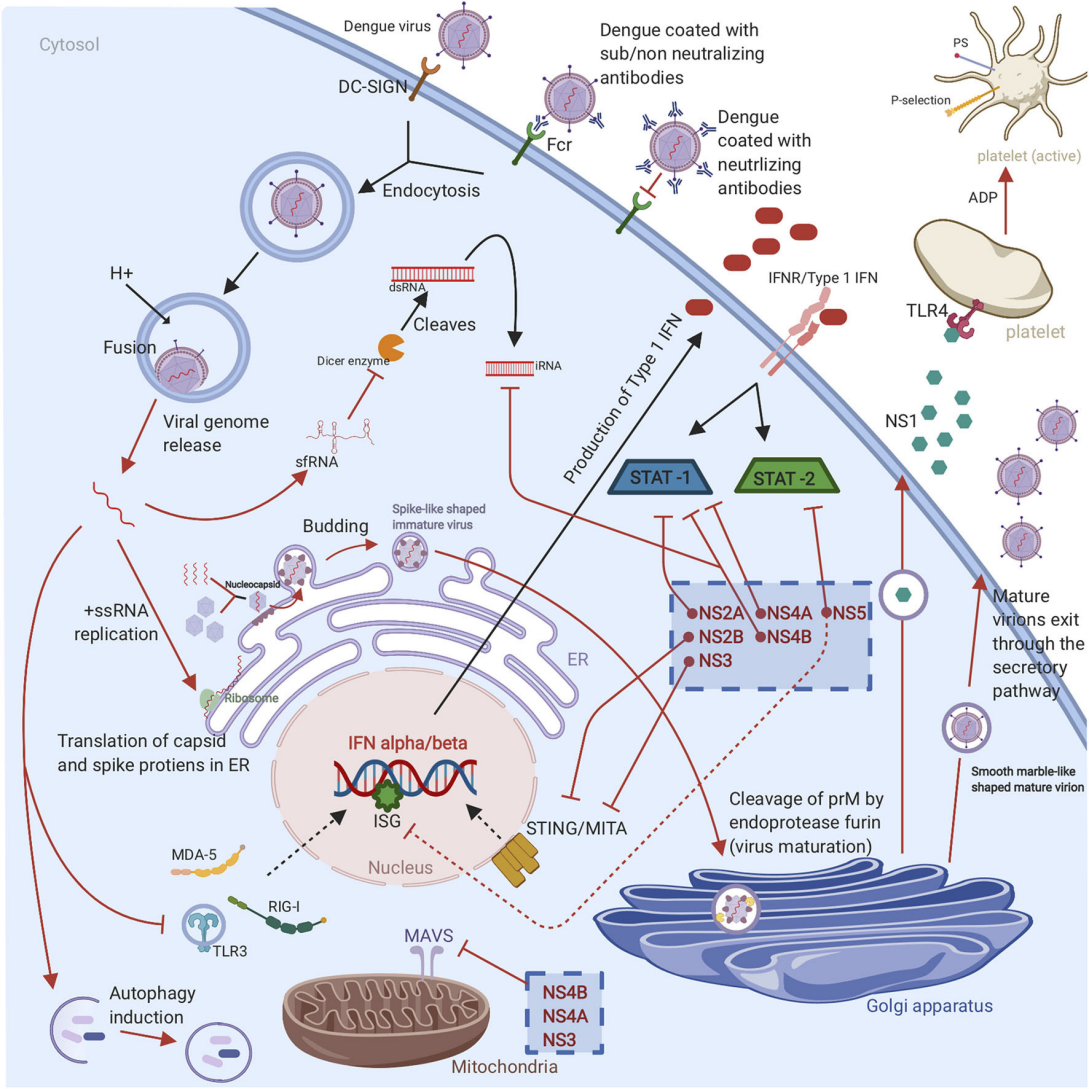
Graphical representation of DENV life cycle and subversion of the innate immune response: The virus enters the host cell through receptor mediated endocytosis or antibody dependent enhancement. Once the virus is endocytosed, the viral RNA escapes the endosome, followed by RNA translation in ER and replication in cytoplasm. Then the newly replicated viral genome is assembled with the C protein to form a nucleocapsid which buds into ER to obtain a lipid membrane coated with prM/M and E proteins. The virion bunds out of ER as immature virion which is characterized by spiky appearance. For further maturation, the virion travel to Golgi apparatus where the prM protein is cleaved by the cellular endoprotease furin to form a mature virion which exists the cell through secretory pathways and infect new cells. The non-structural proteins (NS1, NS2A, NS2B, NS3, NS4A, NS4B, and NS5) of DENV have various evasion mechanisms. These evasion mechanisms include TLR, RIG-I, and MDA5 signaling cascades disruption, suppression of IFN α/β-mediated antiviral responses, MITA/STING cleavage, interference with RNAi response, enhance viral replication by autophagy induction, inhibition the cleavage of double-stranded RNA by Dicer enzyme, suppression the ISGs, inhibition the upstream and downstream of MAVS pathway, STAT1 phosphorylation inhibition, and STAT2 degradation. Finally, the non-structural proteins are able to induce platelet activation, aggregation, and apoptosis that leads to vascular leakage and thrombocytopenia (Red arrows represent various countermeasures that have been developed by dengue to evade and or to hinder antiviral innate immune response. The cellular antiviral response against DENV is indicated with black arrows).

It is important to highlight the significant role of DENV in regulating cellular lipid metabolism and autophagy to enhance replication, maturation, and production of the infectious virions. The mature virions and NS1 hexamers exit the infected cell through the host secretory pathway (13) (Figure 2). It is worth noting that the concentration of secreted NS1 have been shown to be positively corelated with disease severity as high counts of NS1 are associated with DHF and DSS (15). The proposed mechanism is that NS1 binds to platelets via toll-like receptor (TLR) 4, activating the platelets, and induces the expression of the activation marker P-selection and the apoptosis marker phosphatidylserine (PS) on the surface of the platelets. The expression of P-selection on the surface increases the adherence to endothelial cells and the PS exposure triggers phagocytosis by macrophages, which leads to thrombocytopenia in dengue infections (Figure 2). This adhesion to endothelial cells also induces vascular leakage and can cause a cytokine storm (16). NS1 can also enhance platelet aggregation with the presence of adenosine diphosphate (ADP) which is secreted by the activated platelets. Thus, TLR4/NS1 interaction triggers platelet activation, aggregation, and apoptosis (16).

## Immune Response to Dengue Virus

### Innate Immunity

The production of interferons (IFNs) is the first line of defense to DENV that can control early viral replication in target cells (6). Once DENV enters the skin, it is recognized by the pattern recognition receptors (PRRs) such as TLRs and C-type lectin receptors that are expressed on the immune sentinels (8). PRR activation enhances antiviral innate immune responses through activation downstream pathway leading to the production of interferons (IFNs) and tumor-necrosis factor (TNF) (17). TLR-3 and −7 stimulation induces the production of IFN- α and IFN- β. IFN-αβ production play a crucial role in inhibiting DENV infection. The produced IFNs bind to IFN receptors express on cells in an autocrine and paracrine manner. This binding leads to the JAK/STAT pathway activation, hence the production of more than 100 effector proteins (18). All the above-mentioned responses will stimulate DC maturation and B and T cells activation, and consequently, promote the adaptive immune response. However, DENV has developed several strategies to hijack IFN machinery. The NS1 protein is secreted from infected cells as a hexamer into patient's sera. The protease NS2B/3 of DENV has the ability to interfere with IFN α/β induction pathways to downregulate antiviral responses through cleaving the human mediator of interferon regulatory factor 3 (IRF3) activator (MITA or STING) (19). In addition, the non-structural proteins of DENV (NS2A, NS4A and NS4B) can partially block STAT signaling pathway which in turn interfere with IFN signaling between the cells (20) (Figure 2).

Intracellular sensors such as the helicases melanoma differentiation-associated protein 5 (MDA5) and retinoic acid-inducible gene 1 (RIG-I) are also considered to be the first line of defense that are able to recognize the viral RNA and are involved in IFN- β production (13). Both RIG-I and MDA5 are involved in IFN-β production (21). In secondary infections, DENV complexed to non-neutralizing antibodies infects Fc**γ** receptor-bearing cells in a manner known as antibody dependent enhancement (ADE). ADE causes down-regulation of TLR signaling as well as interference with RIG-I- and MDA5-signaling cascades causing the inhibition of the IFN α/β-mediated antiviral response (13). During RIG-I activation, RIG-I will recognize viral RNA and is translocated to the mitochondria where it interacts with an adaptor protein called a mitochondrial antiviral-signaling protein (MAVS). RIG-I/MAVS interaction induces the development of MAVS aggregates, which serve as an immune signalosome that activates the transcription factor IRF3 and nuclear factor kB (NF-κB). Afterward, these transcription factors translocate to the nucleus and induce the production of type I IFN.

DENV has developed evasion strategies to inhibit upstream and downstream from MAVS pathway. DENV protein NS3 is able to prevent the translocation of RIG-I to mitochondria (22). On the other hand, NS4A is able to bind to MAVS CARD domains and effectively prevent RIG-I/MAVS interaction (23). The interference RNA (RNAi) pathway is a vital antiviral response however, DENV has evolved multiple mechanisms to interfere or evade it. The most well-studied of these mechanisms is the generation of a subgenomic flavivirus RNA (sfRNA) from the 3'-untranslated region of the viral RNA (vRNA) (24). The production of sfRNA inhibits the cleavage of double-stranded RNA by the Dicer enzyme to hinder the innate antiviral immunity. Another strategy that has been developed to interfere with RNAi pathway is the expression of the sub-structural protein NS4B which can modulate the host RNAi/miRNA pathway to favor DENV replication (25). The protein NS5 is able to prevent IFN production by suppressing IFN-stimulated genes (ISGs) through inhibiting recruitment of the transcription complex PAF1C (26). As mentioned above, dengue non-structural proteins interplay with innate immunity depicted in Figure 2. It has been shown that the activated mast cell in the skin is the responsible cell for initiating the recruitment of cytotoxic cells including natural killer (NK) cells, natural killer T (NKT) cells, and CD8+ T cells. The recruitment of cytotoxic cells to the site of infection promotes the clearance of virus and limits the infection in the draining lymph nodes (27). In addition to the crucial role of DCs in producing IFNs, TNFs, and blocking the spread and replication of the virus, DCs also link the innate immune response to adaptive immune response by presenting the antigen to T cells after migrates to the draining LNs (8).

### Adaptive Immunity (T Cells)

T cells have been reported to have both pathological and protective function during dengue infection. Dengue-infected DCs present the antigen to CD8+ and CD4+ T cells in the T-cell zones of the draining LN, where the adaptive immune response begins. The activated CD4+ T cells will then provide help to CD8+ T cells that are then able to directly kill dengue infected cells through recognition a variety of dengue proteins including the non-structural NS3 and NS1.2a proteins (8, 28). High numbers of activated CD4+ T cells have been seen in asymptomatic cases in controlling the dengue infection demonstrating its protective role. CD8+ T cells are mostly directed against non-structural proteins whereas CD4+ T cells are skewed toward envelope, capsid, and NS1 epitopes (29). It has been reported that in immune-recall responses to secondary DENV infection the presence of heterologous memory and cross-reactive CD4+ T specific for a primary DENV serotype will exacerbate immune pathology (30).

## Current Dengue Vaccines

### Licensed Vaccine

**Figure 3:**
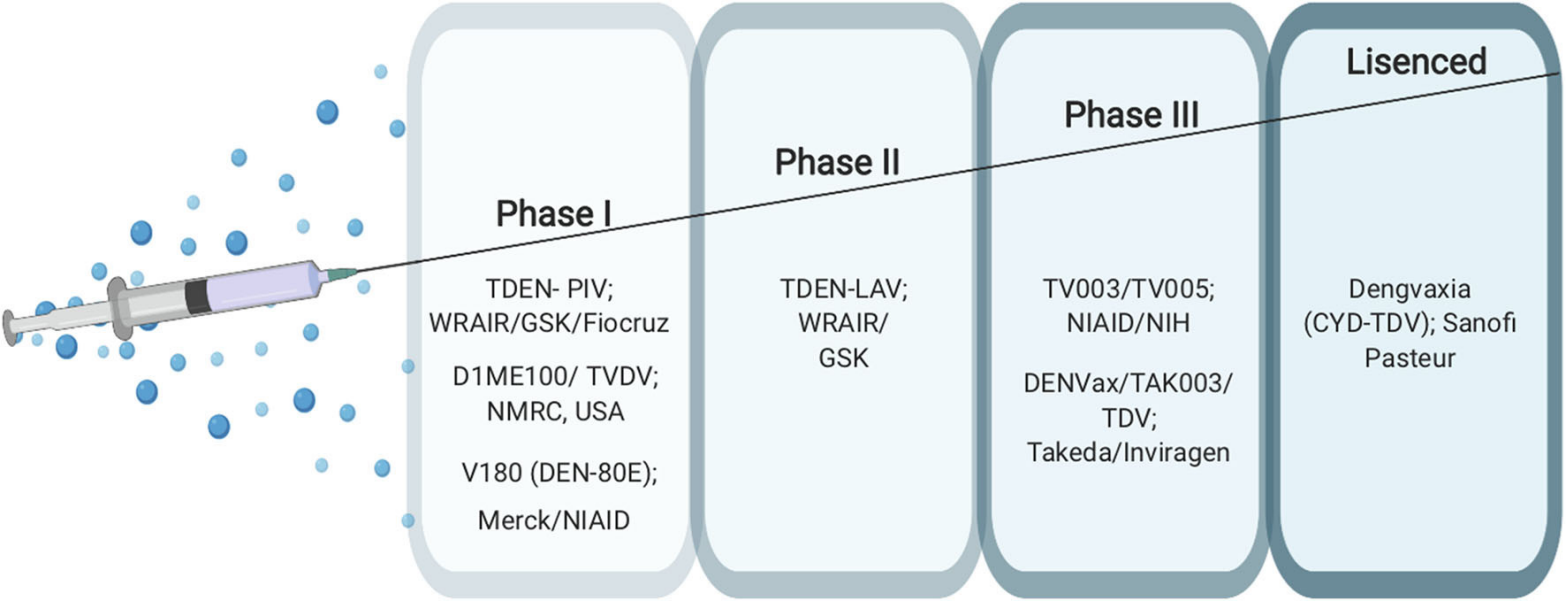
Current dengue vaccines.

Developing an effective vaccine against dengue is challenging due to the fact that the DENV has four serotypes with all four types have the ability to cause disease. In addition, ADE, which is induced by pre-existing antibodies against DENV, creates an obstacle for vaccine development since neutralizing antibodies need to be generated to all serotypes of dengue to confer protection (31). Yet there are several promising dengue vaccine candidates under clinical evaluation (32, 33). So far, Dengvaxia (CYD-TDV) developed by Sanofi Pasteur is the only vaccine licensed and in use in many countries worldwide since 2015 (34, 35). This vaccine is a live attenuated, chimeric, tetravalent vaccine with a Yellow fever 17D strain virus backbone (36, 37). The prM and E proteins of the yellow fever are replaced with the prM and E proteins from the four DENV serotypes (37). This vaccine is licensed to be given only to dengue seropositive individuals with the age group of 9–45 years in dengue endemic countries It is administered subcutaneously in a three-dosage series of 6 months apart (0, 6, 12 months) (35). Despite the fact that CYD-TDV has shown great efficacy in protecting against severe disease in dengue positive individuals, it placed seronegative individuals at an increased risk of developing severe dengue disease (38). For this reason, research to find other possible dengue vaccines is still underway. There are several other vaccine candidates in clinical trials at different advanced stages ranging from Phase I to Phase III. These include live attenuated, purified inactivated and DNA vaccine platforms (39–43) (Figure 3).

### Phase III Vaccines

TV003/TV005 (NCT01506570) and TDV/DENVax/TAK003 (NCT02302066) are two promising live-attenuated vaccine candidates currently ongoing in phase 3 clinical trials (39, 40). The TV003/TV005 vaccine candidate is a live attenuated vaccine developed by The National Institute of Allergy and Infectious Diseases NIAID/NIH (44). This vaccine contains a mixture of four live attenuated dengue serotypes (4). The TV003 vaccine has been shown to induce neutralizing antibodies to all four serotypes in humans. This vaccine contains 10^3^ PFU from each of the four-dengue serotypes 1, 2, 3, and 4 (4). TV005 is identical to TV003 with only a higher dose of 10^4^ PFU of the DENV2 component. Both vaccines have showed promising results in clinical trials with TV003 eliciting the highest robust immune response to all DENV serotypes (DENV1-4) after only a single dose (31, 44). TDV, which is also known as DENVax/TAK003, is a chimeric, tetravalent live attenuated vaccine that was developed by Takeda/Inviragen (NCT01511250). This vaccine consists a chimera of prM and E proteins of DENV1, 3 and 4 serotypes based on a whole live-attenuated DENV2 PDK53 backbone (45). It has shown to induce neutralizing antibody titers against all DENV serotypes and the ability to produce humoral and cellular responses as well (46, 47) (Figure 3).

### Phase II, I, Preclinical Vaccines

TDEN-LAV (NCT01702857) (36) and TDEN-PIV (NCT01666652) (37) are two other vaccine candidates which were developed by the Walter Reed Army Institute of Research (WRAIR) and GlaxoSmithKline Vaccines (GSK). TDEN-LAV is a live attenuated tetravalent vaccine requiring two doses that contains the four serotypes of DENV and is currently in a phase II clinical trial. It has undergone serial passaging in primary dog kidney (PDK) cells and three more passages in fetal rhesus lung cells (FRhL) to reduce infectivity. TDEN-LAV was made in two different formulations termed F17 and F19 with both being tolerated well by healthy adults regardless of their prior priming status with the dengue virus. The F17 formulation produced stable titers for all four serotypes while F19 formulation had loss of infectivity with DENV-4. During the trial, unprimed vaccine recipients did not develop responses to all 4 serotypes after the first vaccine dose yet both formulations elicited immunogenicity across all subtypes after 2 doses. However, the level of neutralizing antibody was not measured and so is unknown.

Live-attenuated vaccine platforms come with one caveat: often, study subjects will develop antibodies against only one dominant serotype rather than all that are included. To combat this problem, it was reasonable to pursue an inactivated dengue vaccine platform. TDEN-PIV (DPIV) is a purified, formalin-inactivated tetravalent DENV vaccine currently in phase I clinical trial (37). DPIV was formulated with either alum or AS01_E_ or AS03_B_ adjuvant systems with two different antigen concentrations. The vaccine regimen includes three doses, one initial dose with two boosters. The study participants were all sero-negative for all four serotypes at the time of vaccine administration. All formulations were well-tolerated by study participants and moderately immunogenic against all four serotypes however there was a waning and a plateau of neutralizing antibodies (48, 49).

Another Phase I vaccine candidate, D1ME100/TVDV, is being developed by the Naval Medical Research Center (NMRC), USA (NCT00290147) (43). It is a monovalent DNA vaccine with a plasmid vector expressing the prM and E genes of DENV-1 under the control of the cytomegalovirus promoter of the plasmid vector VR1012. The vaccine was tested in dengue-naïve participants, and immunogenicity and safety were determined after three doses. DNA vaccines offer several advantages including potent CTL responses and ability to preserve humoral immunity. This is accomplished by producing non-living, non-replicating, and non-spreading antigens that essentially results in mimicking natural infection (50). D1ME100/TVDV induced anti-dengue T cell IFN gamma responses but only 5 of 12 patients that received a high-dose formulation had detectable neutralizing antibody responses that, while long-lasting, were low level (43, 51) indicating that the TVDV vaccine to be safe and favorably reactogenic but without important humoral responses (43).

There are many recombinant subunit vaccine candidates in the vaccine pipeline. V180 (DEN-80E) MERCK (NCT01477580) is one of the most promising vaccine candidates that has completed phase I clinical trial (52). It is an envelope protein-based vaccine containing 80% of the N-terminal of the envelope protein (DEV-80E) for all four DENV serotypes produced using the S2 Drosophila cell line (53). The preclinical trial study used a mixture of this vaccine candidate with ISCOMATRIX™ adjuvant on mice and monkeys to show efficacy in inducing strong neutralizing antibodies against all DENV serotypes and protection against viremia (53, 54). The MERCK phase I clinical trial used flavivirus-naïve adult volunteers who were injected with V180 formulations, including the adjuvant ISCOMATRIX™. Study participants showed a positive robust immunity but formulations with aluminum adjuvant and without adjuvants were poorly immunogenic. The vaccine, when coupled with ISCOMATRIX™, was shown to be associated with more favorable events when compared with formulations with aluminum and non-adjuvanted formulations and overall, all formulations were well-tolerated (55) (Figure 3).

Several other vaccine candidates with different platforms are being tested in preclinical trials with mice and non-human primates including virus-vectored (56–58), recombinant protein (59–61) and virus-like particles (VLPs) (62–64) vaccines but so far, none have made it into phase I trials.

## Pre-vaccine Environment Effect on Vaccine Response

The pre-vaccination microenvironment is poorly understood for vaccine development. There are known and generally well-studied factors that affect vaccine response include age, gender, genetic background, differences in physical environment, and pre-existing immunity. For example, one study looked at the comparison of the response to the licensed yellow fever vaccine YF-17D in healthy adults from different origins and gender. The results show that men of mixed European decent have higher antibody levels when compared with females of the same decent, or individuals of African descent or Hispanics (65). Recently, researchers from Oxford published a study outlining just how genetic variation can affect vaccine response and the persistence of immunity after childhood vaccinations. They detail the considerable variability in the magnitude and persistence of vaccine-induced immunity due to genetic factors using genome-wide association study (GWAS) in the childhood vaccines capsular group C meningococcal (MenC), Hemophilus influenzae type b, and tetanus toxoid (TT) vaccines. In doing so, they were able to define associations between the single nucleotide polymorphisms (SNPs) in the human leukocyte antigen (HLA) locus and the persistence of immunity (66). Aging has also been shown to play a large factor in vaccine response. For example, two large-scale clinical trials compared the highly successful yellow fever vaccine YF-17D immunogenicity between adults and elderly individuals. One found no difference between the generation of neutralizing antibodies between the two groups, but the other trial found that the elderly cohort had a delayed antibody response and higher viremia (67).

Pre-existing immunity or “original antigenic sin” is a well-known barrier to a productive vaccine especially for flaviviruses which are all antigenically related. This phenomenon can modulate immune response to sequential infections or vaccinations. In general, the immune memory to cross-reactive antigenic sites and the formation of immune complexes can affect antibody responses in any sequential infections or immunizations with similar antigens. This was shown in a recent paper in 2019 where pre-existing yellow fever immunity from infection impaired the antibody response to the tick-borne encephalitis vaccination (68). It is important to consider this prospect especially in dengue vaccine development.

Nevertheless, the pre-vaccine microenvironment, like the levels of inflammation and immune activation that is already active in an individual, has a great impact on how a patient will respond to a particular vaccine. In a study published in 2014, researchers looked at the pre-vaccination environment and vaccine responses between study participants from either Entebbe, Uganda or Lausanne, Switzerland that were vaccinated against yellow fever with the licensed yellow fever vaccine YF-17D. They found fundamental differences in the subsequent cellular or humoral responses after vaccination including a substantially lower CD8+ T cells and B cells from the Entebbe cohort compared with immunized individuals from Lausanne meaning an impaired vaccine response. The researchers also observed higher frequencies of differentiated T and B cell subsets, exhausted and activated NK cells, and proinflammatory monocytes suggesting that an activated immune microenvironment in the Entebbe cohort prior to vaccination led to differences in vaccination responses. The activation of the proinflammatory monocytes at baseline resulted in a negative correlation with YF-1D neutralizing antibody titers after vaccination (69). Though we have known that aging plays a role in how a subject will respond to a vaccination, it is only recently that the mechanisms have been researched. Researchers in 2015 reported that the pre-vaccination inflammation and blunted B cell signaling due to aging correlates with the hyporesponse to the hepatitis B (HBV) vaccination (70). Specifically, using transcriptional and cytometric profiling of whole blood collected before vaccination, they show that there is an increase in inflammatory response transcripts and pro-inflammatory monocytes in the older cohort that correlates with poor vaccine response to the HBV vaccine. Conversely, augmented B cells responses and a higher frequency of B cells correlated with a stronger response to the vaccine in the younger individuals. This study was the first to identify baseline responses that could predict responses to the HBV vaccine and possibly others. Therefore, the existence of the pre-vaccine immune microenvironment should be taken into consideration for the development of any vaccine.

## Challenges Face Dengue Vaccine Development

### Antibody Dependent Enhancement (ADE)

**Figure 4:**
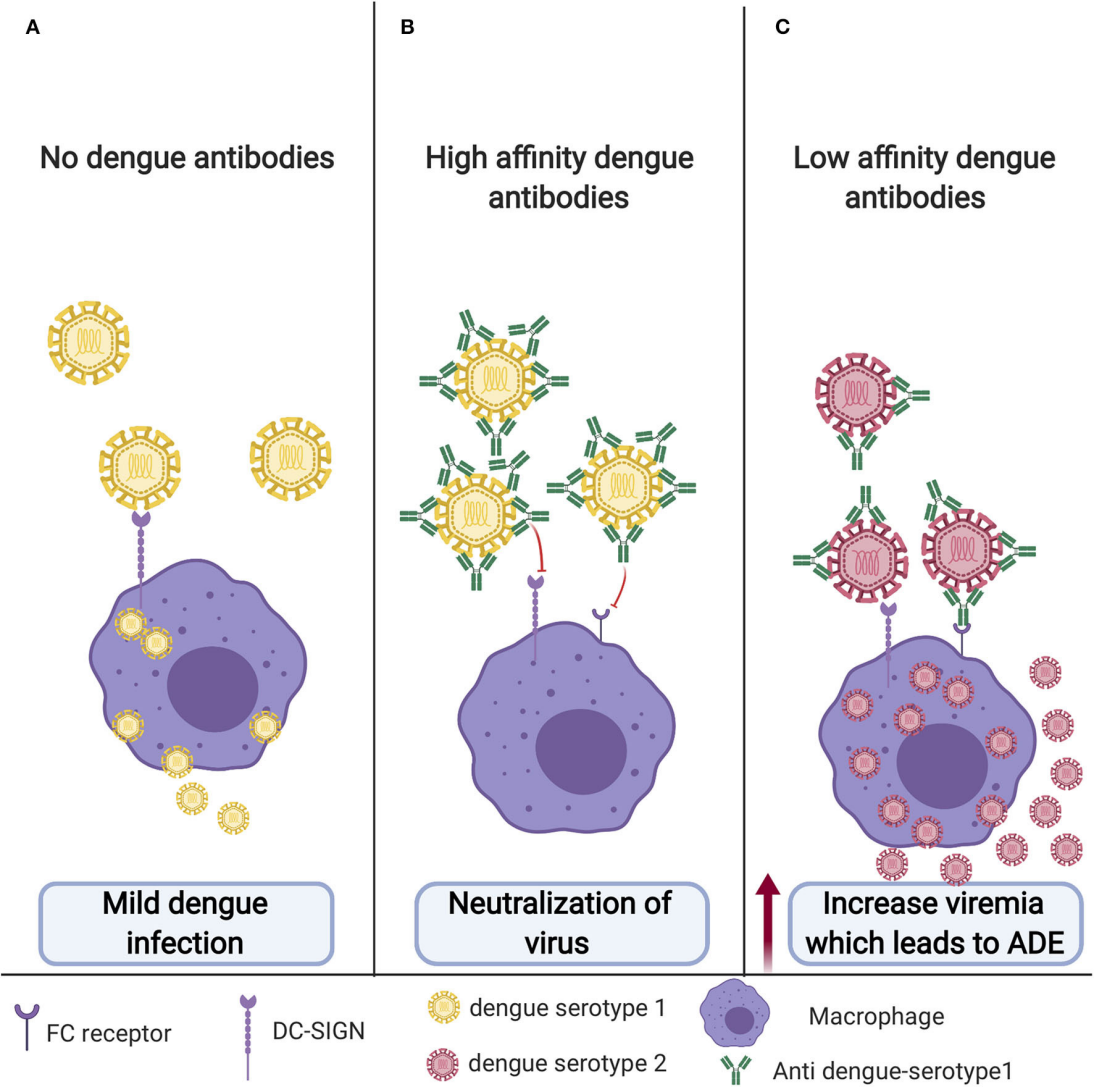
Antibody dependent enhancement (ADE): **(A)** Primary infection with no previous vaccination. DENV will enter macrophage through its cognate receptor; however, most of the time, it will result in mild disease, and sometime this could even be symptomatic or with mild flu-like symptoms. **(B)** DENV in the presence of neutralizing antibodies for the same serotype. DENV will not be able to enter the cells and establish infection. **(C)** Cross-reactive antibodies from previous unsuccessful dengue vaccine or dengue infection with different serotypes will bind but not neutralize the virus. This low-affinity binding will facilitate the entry of the virus to the macrophage through FC receptor resulting in increased viremia leading to ADE.

Unlike other highly effective vaccines developed against other flaviviruses, the development of a dengue vaccine is highly challenging due to that fact that the virus has four antigenically different serotypes (DENV1–4). For an ideal dengue vaccine, the vaccine should be effective against all four serotypes at the same time. Primary DENV infections are usually asymptomatic or with mild flu like symptoms (Figure 4A). Post DENV infection it takes antibodies ~1 week to develop. During primary infection with one DENV serotype, antibodies produced by this serotype usually results in a long-lasting protection against that particular serotype and short lived protection against other serotypes (5). Antibodies play a dual role in controlling DENV infection, in which they can either neutralize or enhance the entry of the virus (4). A study that analyzed antibodies produced in human post-primary DENV infection found low amounts of highly specific and neutralizing antibodies that were mainly against the envelope EDIII domain. On the other hand, they found that most weakly cross-reactive antibodies were against prM (71). Preexisting neutralizing antibodies can prevent DENV attachment to its natural receptor on the cell surface thus inhibiting virus entry (Figure 4B). However, antibodies from heterologous infection can be cross-reactive and facilitate a process known as antibody dependent enhancement or ADE. This mechanism allows the virus to enter and escape the endosome and go through a manner similar to the primary infection pathway causing a higher virus burden and ultimately enhancement of disease (5, 72). ADE has been observed for a variety of viruses including HIV, Ebola, and possibly the virus responsible for the recent pandemic, SARS-CoV2. Fc receptor (FcR)-dependent ADE is accepted as the most common mechanism of ADE among many viruses, including dengue, HIV, and influenza A. Virus-antibody complexes will bind to cells that have a FcR like macrophages, monocytes, B cells, and neutrophils through the interaction between the Fc portion of the antibody and the FcR on the cell surface. This essentially creates an immune synapse that increases the attachment of viruses to the cells (73) (Figure 4C). Another possible mechanism of ADE involves the activation of the complement classical pathway. While FcRs are only expressed on immune cells, complement receptors (CRs) are broadly expressed on most cells (74). For example, HIV ADE can occur via FcR or by virus-C3 fragment complexes and classical CRs that will facilitate normal virus entry by viral surface protein gp120 and its receptors and coreceptors (75). Additionally, Ebola utilizes another complement mediated ADE mechanism in in which antibodies bind in proximity, allowing C1q to bind to the Fc portion of the antibodies. This complex (virus, antibodies, and C1q) binds to C1q receptors (C1qR) which facilitates either endocytosis or binding of the virus to Ebola-specific receptors (74, 76). Recently, Wan et al. published an ADE mechanism in Coronaviruses. Their results indicate that ADE of coronaviruses may be mediated by neutralizing antibodies that target the receptor binding domains of the coronavirus spikes. Interestingly and unlike dengue that involves ADE with the different serotypes, the same coronavirus strains that produce fully neutralizing antibodies can be mediated to go through ADE by the same neutralizing antibodies (77). It is also unclear as to whether virus-specific receptors are required for ADE entry. It may depend on whether the virus is enveloped or non-enveloped and the mechanism of ADE but if a virus relies on surface receptors only for binding, the virus may be able to infect the cells via FcR without a natural receptor. This models how non-susceptible cells that do not express a virus's natural receptor can be infected when FcR is expressed like in FcR-mediated ADE of foot and mouth disease (78).

The reason behind the high number of infected cells and high viral particles following ADE have been shown in a study in which DENV-immune complexes can suppress the antiviral immune response by down regulating the production of IL-12, TNF-a, IFN-γ, and nitric oxide radicals (NO), and enhancing the expression of IL-6 and IL-10, thus promoting virus particle production (79). ADE occurs in dengue-infected individuals who previously had been infected with different serotype from the first one or other flavivirus. ADE could also occur upon poor response to vaccination. Both anti-E and anti-prM antibodies have been shown to enhance DENV entry into the target cells through Fc**γ**- mediated ADE (4). A study published in 2010 suggested that response toward cross-reactive epitopes such as prM could be a part of the immune evasion mechanism by DENV. Furthermore, they have advised the reduction of anti-prM response in dengue vaccine design to reduce ADE (80). One example of a DENV vaccine candidate that steered away from prM is a preclinical vaccine. This vaccine is VLP based designed to produce antibodies against the EDIII domain and has demonstrated decreased ADE in animal models (62). Additionally, a recent study in 2019 using molecular simulations found that higher cross-reactive DENV antibodies were linked to higher ADE and that poorly immunogenic vaccine enhances ADE (81). Ultimately, ADE is the main causative factor in the progression of the self-limited dengue fever to DHF and DSS (4, 82–84) (Figure 4).

### Cross Reactivity With Other Flaviviruses

There are several challenges that have hindered the development of the dengue vaccine. One of these challenges is the structural similarities between DENV and other members of the Flaviviridae family viruses such as the Zika virus (ZIKV), Yellow fever virus (YFV), Japanese encephalitis virus (JEV) and West Nile virus (WNV). The envelope protein (E) is both structurally conserved among flaviviruses and the most exposed protein to which the immune system generates antibodies against in order to neutralize the virus. The E protein consists of three functionally and structurally distinct domains EDI, EDII, and EDIII (85). The envelope protein (E) of DENV shares more than 50% homology with the ZIKV E protein, resulting in cross reactivity (86). The cross reactivity contributes to either protection or pathogenic enhancement to a second infection with one of the members depending on the quantity and the specificity of the generated antibodies. In 2016, ZIKV outbreaks overlapped the regions where the DENV was endemic in the north of Brazil and Mexico (85, 87). Consequently, the individuals that have been infected by ZIKV were likely to be pre-exposed to DENV and vice versa. This created a concern among researchers since preexisting immunity to other flaviviruses affects immune responses induced by DENV which may result in severe dengue manifestation.

Studies show that both humoral and cellular immunity contribute to disease pathogenesis with humoral immunity being the main causative factor of ADE. However, high concentrations of pre-existing cross-reactive antibodies have been found to have the ability to reduce the probability of symptomatic dengue infections (88). Therefore, the threshold of cross-reactive antibody concentration must be reached to effectively neutralize and inhibit virus attachment and entry. On the other hand, if the cross-reactive antibody titers do not reach the threshold, ADE occurs, and the neutralization fails. One recent study done within the Mexican population determined the response of cross-reacting antibodies in the sera of patients with DENV against the recombinant envelope protein of ZIKV (85). They demonstrated that the serum samples of the dengue-infected patients have cross-reactive antibodies against the E protein of ZIKV which can either mediate ADE or neutralize the infection depending on the concentration of the antibodies (85). It has been observed that the protection against severe infections lasts for 2 years following the primary infection after which the neutralizing antibodies decay and the risk of symptomatic and severe dengue infection increases upon secondary heterologous infection (88). There is some evidence that a simultaneous re-exposure is required to maintain the cross-reactive neutralizing antibodies for a longer time (89). Most of the effectual vaccines provide protection against pathogens by generating neutralizing antibodies. Long-lived antibody-secreting plasma cells are produced by the germinal centers (GC) that are formed in the secondary lymphoid tissues with the help of T follicular helper cells (Tfh) (90). Harnessing this mechanism for long-lived antibody secreting plasma cells is vital for a thoroughly effective dengue vaccine.

## Targeting Tfh Cells to Enhance Dengue Vaccine Efficacy

**Figure 5:**
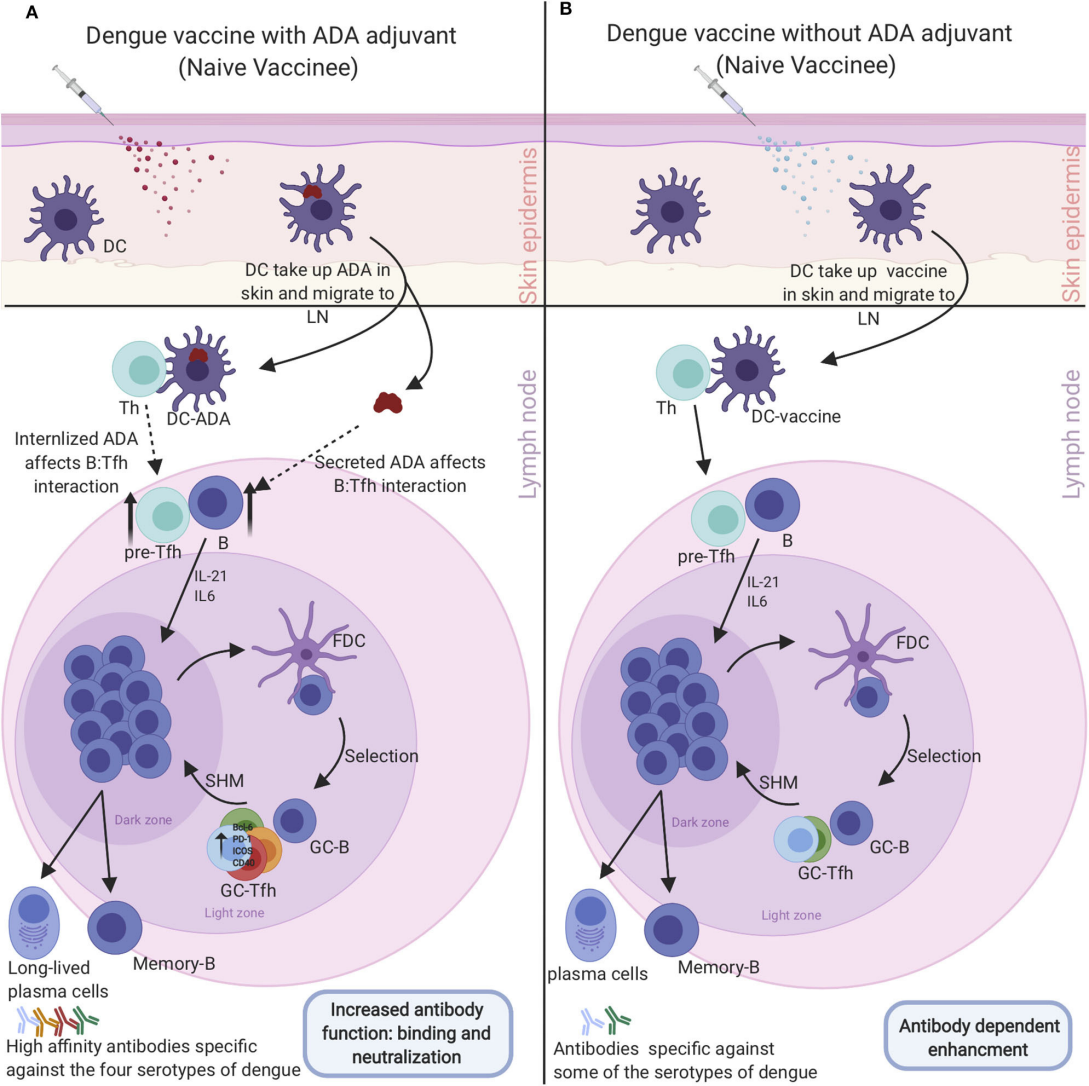
Enhancement of dengue vaccine by targeting Tfh cells to overcome dengue serostatus effect: **(A)** Administering dengue vaccine to naive dengue individual with Tfh Adjuvant (ADA) will be taken up by DC that travels to the LN and prompt enhanced Tfh-B cell interaction. The enhanced interaction induces Tfh-specific cells to the four dengue serotypes leading to differentiation of memory B cells and plasmablasts that produce high-affinity neutralizing antibodies against all the serotypes which neutralize the dengue virus. **(B)** Administrating dengue vaccine to naive individuals without Tfh adjuvant (ADA). Vaccine will be taken up by DC, which will travel to the LN and induce medium Tfh-B cell interaction. The medium interaction will give rise to Tfh, B cells, and plasmablasts that are specific to some serotypes of dengue. Antibodies produced by this response will make them vulnerable to ADE and enhanced dengue diseases upon infection with a different dengue serotype virus that they have sub-neutralizing antibodies against.

Germinal center (GC) responses are supported by a specialized type of CD4+ T cells called Tfh cells. Tfh are mainly located in the GC, however, counterparts of these cells are present in the peripheral blood which can be identified by expression of CXCR5, ICOS, and PD-1 (91, 92). There are growing interest in studying circulating peripheral blood Tfh (cTfh) instead of GC Tfh and using them as biomarkers of GC activity since collecting a healthy human lymphoid tissue can be more difficult than peripheral blood (93, 94). CTfh cells come in different subtypes with each expressing different cytokines and therefore having different abilities to provide help for the B cells (95, 96). CTfh1 are mostly considered as the inefficient helper while cTfh2 and cTfh17 are the efficient helper subtypes. Furthermore, these cells have been highly correlated with broadly neutralizing antibodies (95, 97). These cTfh cells provide a great tool for monitoring vaccine responses. Generally, Tfh activate GC B cells by producing IL-21 and up-regulating various proteins and transcriptional factors such as ICOS, Bcl-6, PD-1, and CD40 (98). Antigen-activated B cells migrate to the B cell follicle in the secondary lymph tissue where they differentiate, proliferate, and undergo through class switching, somatic gene hypermutation (SHM) and, affinity maturation. B cells that have been through SHM exit the division cycle to test their recently mutated B cell receptor by interacting with the antigens expressed by the antigen-presenting cell follicular dendritic cells (FDC). Finally, the B cells must undergo the selection process to exit the GC as long-lived plasma cells and durable memory cells. The selection process occurs by presenting the processed antigen on B cells to Tfh cells to select B cells with higher affinity for the pathogen (98). A recent study showed an increased activation of the Tfh cells in the critical phase of illness compared to mild and moderate phase of illness that was highly correlated with high frequency of plasma blasts. Furthermore, the number of activated peripheral Tfh in secondary DENV infections is increased compared with primary DENV infections (99). This might be due to the activation of Tfh cells specific only for one serotype resulting in ADE and disease pathogenesis. However, we hypothesize that enhancing Tfh cells specific to all serotypes would solve ADE. Eventually, Tfh cells support the GC response and positively regulate the magnitude of the GC response. Using Adenosine deaminase-1 (ADA-1) as an adjuvant has been shown to be one of the potential strategies to modify and enhance Tfh function. ADA-1 is an intracellular enzyme which converts adenosine into inosine through the deamination process. ADA-1 is also involved in the development and maintenance of the immune system by potentiating the differentiation of naive T cells to effector, regulatory, and memory CD4+ T cells (100). It has a central role in the immune system as mutation of ADA-1 leads to severe immunological disorders and loss of functional T, B, and NK cells (101). One study, using PBMCs and tonsil cells from HIV-infected patients, shows that ADA-1 is essential for an efficient GC-Tfh response and promotes antibody affinity maturation within the GC by providing a favorable cytokine microenvironment (102). Many studies have shown a strong correlation between efficient induction of memory B cells and plasma cells that will produce specific neutralizing antibodies against influenza and Ebola and increasing Tfh cells in the context of the immunizations (93, 97, 103). It is important to identify potential adjuvants that will efficiently target and induce a Tfh response for future vaccine design. Most vaccines depend on adjuvants to improve the immune response, increase neutralizing antibody titers, induce long-lasting immunity, and reduce required vaccine doses. In the context of Tfh induction, water-in-oil adjuvants have been shown to selectively promote the Tfh response, such as incomplete Freund's adjuvant (IFA), Montanide ISA 720, and ISA-51 (104). Another study showed the MF59 oil-in-water adjuvant mediates a potent Tfh response that directly promotes GC responses (105). Other adjuvants such as TLR4, TLR6, TLR7, TLR8, and TLR9 agonists had extensive interest in the use of vaccine adjuvants as TLR agonists can all enhance Tfh cells (106). The Tfh cells have to be selectively and potently enhanced to overcome the seronegative group setback in dengue vaccination in order to generate specificity to all 4 serotypes concurrently using a potent adjuvant such as ADA (Figure 5). On the other hand, enhancement of Tfh activity has been linked with multiple autoimmune diseases such as Systemic Lupus Erythematosus (SLE), Rheumatoid Arthritis (RA) and, multiple sclerosis (MS) (107–110). In conclusion, Tfh cells are a double-edged sword and transient enhancement of their activity would be beneficial for the development of a precise dengue vaccine that would generate neutralizing antibody titers to all dengue serotypes regardless of vaccines dengue serostatus.

## Summary

DENV is a significant health concern and the development of the best vaccine possible is needed to decrease the burden of this disease on society. Dengue is a very tricky and challenging virus because it has four separate dengue serotypes. That means that in order to design an effective dengue vaccine, it has to induce neutralizing high-affinity antibodies to the 4 serotypes simultaneously to avoid ADE. So far, the only licensed dengue vaccine Dengvaxia (CYD-TDV), developed by Sanofi Pasteur, taught us a vital lesson that dengue serostatus affects vaccine response. With Dengvaxia, dengue naïve individuals did not respond appropriately to the vaccine compared to immune individuals. This difference between the two groups needs to be investigated at the prevaccination microenvironment level to address this issue. However, we speculate that the low activation of Tfh cells, specific to each of the four serotypes, is the fundamental difference between the two groups. This issue could be addressed by adding adjuvants such as ADA that potently activate the Tfh cells and give rise to Tfh specifics to the 4 serotypes of the virus. We believe this could make the naïve individuals respond to the vaccine and give rise to high-affinity neutralizing antibodies to all the 4 serotypes and make them respond as well as dengue immune vaccinated individuals.

## Author Contributions

SanA, SawA, and JC contributed to writing sections of the paper and figure design. EH and AI contributed to the concept, structure, and writing up the paper. All authors contributed to the article and approved the submitted version.

## Conflict of Interest

The authors declare that the research was conducted in the absence of any commercial or financial relationships that could be construed as a potential conflict of interest.
